# Epitaxial Growth of Crystalline CaF_2_ on Silicene

**DOI:** 10.1021/acsami.2c06293

**Published:** 2022-07-06

**Authors:** Daniele Nazzari, Jakob Genser, Viktoria Ritter, Ole Bethge, Emmerich Bertagnolli, Tibor Grasser, Walter M. Weber, Alois Lugstein

**Affiliations:** † Institute of Solid State Electronics, Technische Universität Wien, Gußhausstraße 25-25a, 1040 Vienna, Austria; ‡ Infineon Technologies Austria AG, Siemensstraße 2, 9500 Villach, Austria; § Institute for Microelectronics, Technische Universität Wien, Gußhausstraße 27-29, 1040 Vienna, Austria

**Keywords:** silicene, CaF_2_, 2D materials, 2D-FET, epitaxy

## Abstract

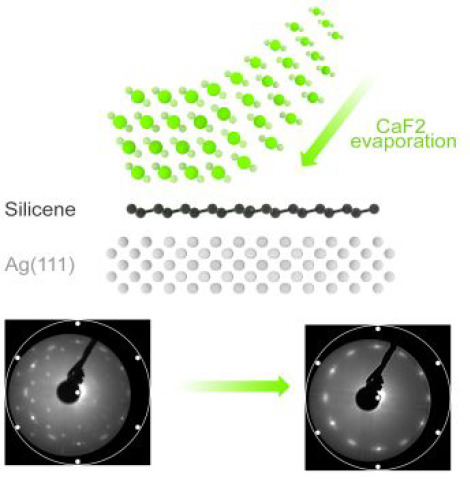

Silicene is one of the most promising two-dimensional (2D) materials for the realization of next-generation electronic devices, owing to its high carrier mobility and band gap tunability. To fully control its electronic properties, an external electric field needs to be applied perpendicularly to the 2D lattice, thus requiring the deposition of an insulating layer that directly interfaces silicene, without perturbing its bidimensional nature. A promising material candidate is CaF_2_, which is known to form a quasi van der Waals interface with 2D materials as well as to maintain its insulating properties even at ultrathin scales. Here we investigate the epitaxial growth of thin CaF_2_ layers on different silicene phases by means of molecular beam epitaxy. Through electron diffraction images, we clearly show that CaF_2_ can be grown epitaxially on silicene even at low temperatures, with its domains fully aligned to the lattice of the underlying 2D structure. Moreover, in situ X-ray photoelectron spectroscopy data evidence that, upon CaF_2_ deposition, no changes in the chemical state of the silicon atoms can be detected, proving that no Si–Ca or Si–F bonds are formed. This clearly shows that the 2D layer is pristinely preserved underneath the insulating layer. Polarized Raman experiments show that silicene undergoes a structural change upon interaction with CaF_2_; however, it retains its two-dimensional character without transitioning to a sp^3^-hybridized silicon. For the first time, we have shown that CaF_2_ and silicene can be successfully interfaced, paving the way for the integration of silicon-based 2D materials in functional devices.

## Introduction

Epitaxial two-dimensional (2D) materials have attracted a high level of interest, as they possess unprecedented optical and electronic properties and their synthesis process is easily scalable.^1^ Among these, silicene is considered to be a particularly attractive candidate^2,3^ for the realization of next-generation high-performance electronic devices, as it is characterized by an ultrahigh carrier mobility^4^ and a band gap that can be tuned by the application of a perpendicular electric field.^5,6^ Silicene is also expected to show intrinsic topological properties^7^ and host Dirac cones at the K points of the Brillouin zone.^8^

In order to engineer, for example, a silicene-based field effect device, it is necessary to develop a gating interface that couples with the 2D layer without altering its bidimensional nature and that is capable to withstand high-intensity electric fields.

Al_2_O_3_ has been successfully employed to passivate silicene, acting as gate insulator in the first silicene-based field effect transistor (FET).^3^ However, in the framework of 2D-FETs, crystalline insulators, such as layered hexagonal boron nitride (hBN) or ionic crystals, like CaF_2_, are expected to offer better performances compared to classic oxides.^9,10^ This advantage stems from their characteristic of having an inert surface, free of dangling bonds,^11^ a prerequisite for preserving the properties of encapsulated 2D semiconductors and for strongly reducing the density of traps at the interface.^12^ Additionally, crystalline insulators possess much narrower defect bands compared to amorphous oxides, which reduces the number of border traps.^13^

Epitaxially grown calcium fluoride—CaF_2_—is especially promising: it possesses a high dielectric constant (ε = 8.43) and a wide band gap (*E*_g_ = 12.1 eV), and its crystal structure is fluorine-terminated along the (111) direction, resulting in a completely inert surface.^14,15^

It has been shown that a high-quality, single-crystal CaF_2_(111) layer can be grown at relatively low temperatures of ∼250 °C on Si(111) by molecular beam epitaxy (MBE), thanks to the extremely small lattice mismatch (<1%) with the substrate.

This moderate growth temperature is important to achieve pinhole-free films with better insulating properties,^16^ while remaining below the thermal stability limit of silicene.^17^ The obtained CaF_2_ is characterized by an extremely low concentration of defects, allowing layers with a thickness of just 2 nm (equivalent oxide thickness of 0.9 nm) to be able to withstand high electric fields (up to 27.8 MV/cm), with negligible leakage currents.^10,18,19^

Crystalline CaF_2_ was recently successfully employed for the realization of MoS_2_-based FET, with high on/off current ratios up to 10^7^ and small hysteresis.^10^

In this study, we demonstrate the successful epitaxial growth of a thin layer of crystalline CaF_2_ on one monolayer (ML) of silicene. A low-energy electron diffraction (LEED) analysis has proven that CaF_2_ grows epitaxially on the 4 × 4, √13 × √13 R13.9° and 2√3 × 2√3 R30° phases of silicene on Ag(111), strictly following the orientation of 2D silicon domains. Furthermore, by monitoring the Si 2p core level via in situ X-ray photoelectron spectroscopy (XPS), we demonstrate that the Si atoms of the buried silicene layer are not forming covalent bonds with either Ca or F atoms. Finally, the vibrational properties of silicene are analyzed by polarized Raman spectroscopy, evidencing a structural modification of the 2D layer. The encapsulated silicene retains its bidimensional nature. However, a clear shift of the main vibrational modes hints at an increased buckling and Si–Si bond length. In conclusion, here we demonstrate that crystalline CaF_2_ can be epitaxially grown on silicene, paving the way for the realization of an ultrathin gate insulation layer in silicene-based electronic devices.

## Discussion

**Figure 1:**
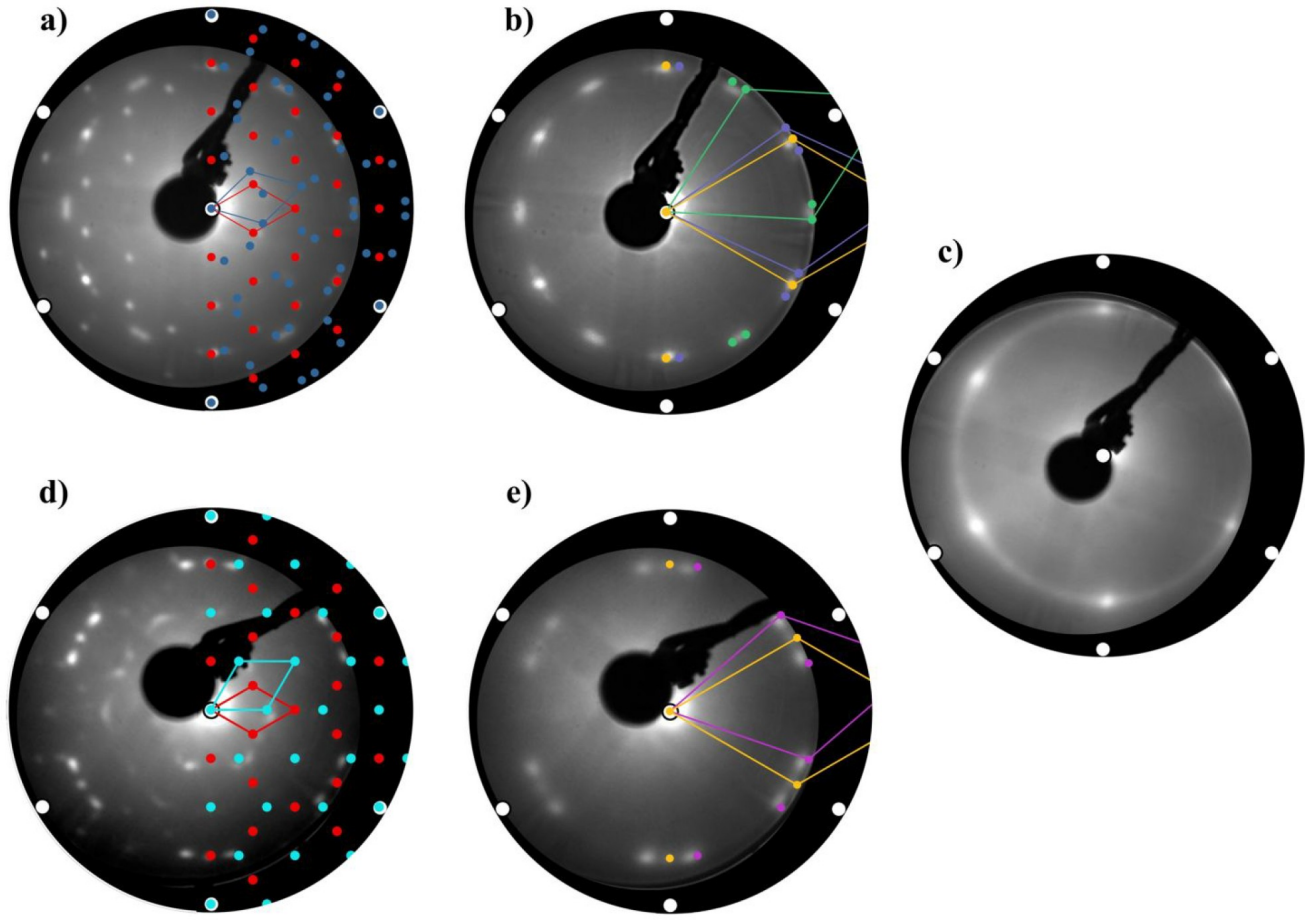
LEED patterns acquired at an energy of the primary electron beam of 35 eV. In all the images, the white disks represent the position of the first-order diffraction spots of the Ag(111) growth substrate. The shown patterns correspond to (a) one ML of silicene grown on Ag(111) at 260 °C, composed of a 4 × 4 phase (red) and of a √13 × √13 R13.9° one (blue); (b) 1 nm thick layer of CaF_2_ deposited on top of the silicene layer shown in (a). Different CaF_2_ domains are visible, oriented with an angle of 0° (yellow), ±5.2° (purple), ±33° (green), with respect to the Ag[10] direction; (c) 1 nm thick layer of CaF_2_ deposited on the bare Ag(111) substrate; (d) one ML of silicene grown on Ag(111) at 300 °C. The pattern is composed of a 4 × 4 phase (red) and of a 2√3 × 2√3 R30° one (turquoise). (e) 1 nm thick layer of CaF_2_ deposited on the silicene layer shown in (d). CaF_2_ domains are oriented with an angle of 0° (yellow) and 10.9° (pink), with respect to the Ag[10] direction.

Figure 1a shows the LEED pattern of one ML of silicene on Ag(111) grown at a substrate temperature of 260 °C. The diffraction pattern can be well-interpreted assuming two silicene phases that are expected for this rather low growth temperature,^20−22^ as shown by the superimposed diffraction model in the right hemisphere. The red pattern is related to the formation of a 4 × 4 silicene phase, while the blue signal reflects the diffraction spots related to the √13 × √13 R13.9° one. In both cases, the Wood notation describes the size and orientation of the silicene supercells with respect to the Ag(111) lattice. Here and in all other images of Figure 1, the white circles represent the position of the first-order diffraction spots of Ag(111). Overall, these supercells account for a total of five different orientations of the silicene layer on top of the Ag(111) substrate. Specifically, the 4 × 4 one accounts for a structure where the silicene[10] vector is aligned along Ag[10] (i.e., vector on the Ag(111) plane), while the √13 × √13 R13.9° supercell comprises four different silicene domains, where the angle between the two vectors is either ±5.2° or ±33°, as clearly demonstrated by Resta et al.^22^

Figure S1 shows a graphical representation of the silicene phases on Ag(111) to better visualize the geometrical orientations of the domains.

After the LEED pattern is acquired, the CaF_2_ evaporation is ramped up, and ∼1 nm of CaF_2_ is deposited on top of the silicene layer, at the same growth temperature of 260 °C.

The relatively low sample temperature for the CaF_2_ growth was chosen for two reasons: first, it has been reported that CaF_2_ layers grown at this temperature are pinhole-free;^23^ second, the growth temperature must be low enough to preserve the silicene layer, which is known to degrade at temperatures higher than ∼325 °C.^24^

What is immediately noticeable when comparing the LEED pattern before (Figure 1a) and after (Figure 1b) CaF_2_ deposition is the markedly reduced number of diffraction spots.

This pattern, however, can be easily modeled by assuming the presence of different CaF_2_ domains, with several orientations with respect to Ag(111). As shown in the right half of Figure 1b, it is therefore possible to clearly identify domains with a rotation of 0° (yellow), ±5.2° (purple), and ±33° (green) with respect to Ag[10]. These orientations are exactly the same ones observed for the silicene layers (i.e., the silicene honeycomb structure and not the supercells, as shown in Figure S1),^22^ suggesting that the growth of the CaF_2_ domains is precisely aligned with the underlying silicene phases.

If the same quantity of CaF_2_ is deposited directly on a bare Ag(111) substrate, the observed diffraction pattern is drastically different, as shown in Figure 1c. In this case, the diffraction pattern is composed by only six diffraction spots associated with CaF_2_ domains aligned with the Ag(111) substrate and a bright diffraction ring. This indicates that the CaF_2_ layer grown directly on Ag is highly polycrystalline and that only a little fraction of the domains is aligned with the substrate lattice, while the majority is randomly distributed. The high content of randomly oriented domains is likely due to the large lattice mismatch (>25%) between CaF_2_ and Ag.

It is well-documented that, by increasing the growth temperature to 300 °C, it is possible to obtain an additional silicene phase, described by the supercell 2√3 × 2√3 R30°, as shown in Figure 1d, corresponding to a 2D lattice rotated by ∼10.9° with respect to Ag[10].^20,25^

As shown in the right hemisphere of Figure 1d, the diffraction spots can be well-modeled by taking into account the high-temperature silicene phase (turquoise), alongside the already mentioned 4 × 4 phase (red). Figure 1e shows the diffraction pattern with the 1 nm thick CaF_2_ layer atop, which was grown again at 260 °C. The diffraction pattern can be well-modeled by considering CaF_2_ domains with an angle of 0° (yellow) and 10.9° (pink) with respect to the Ag[10] direction, matching again perfectly the orientation of the underneath silicene phases. Thus, it is clear that CaF_2_ grows epitaxially on silicene or in other words that the presence of just a single layer of silicene is able to direct the growth of CaF_2_. By taking into consideration several diffraction patterns of CaF_2_ on silicene, we measured the lattice constant of the deposited material, obtaining a value of *a*_CaF_2__ = 0.55 ± 0.01 nm, matching the value reported in literature.^26^

The absence of silicene-specific spots does not mean that the crystalline structure of silicene is lost: LEED cannot probe the buried 2D layer, a direct consequence of the extremely small inelastic mean free path of the electrons employed in this technique.^27,28^ It rather indicates that the 1 nm thick CaF_2_ layer completely covers the silicene layer.

Such a precise correspondence between the CaF_2_ domains and the silicene layer might be a sign of the formation of covalent bonds between Si and Ca or F atoms, or it may be driven by the energetically favorable alignment of their respective lattices due to the extremely small lattice mismatch between silicene and CaF_2_ (<1%).

To obtain more precise information on the silicene/CaF_2_ interface properties, the sample is analyzed using XPS, before and after CaF_2_ deposition. From now on, all investigations will refer to the silicene layer obtained at 260 °C, as the high-temperature phase is known to be highly defective.^29^

**Figure 2:**
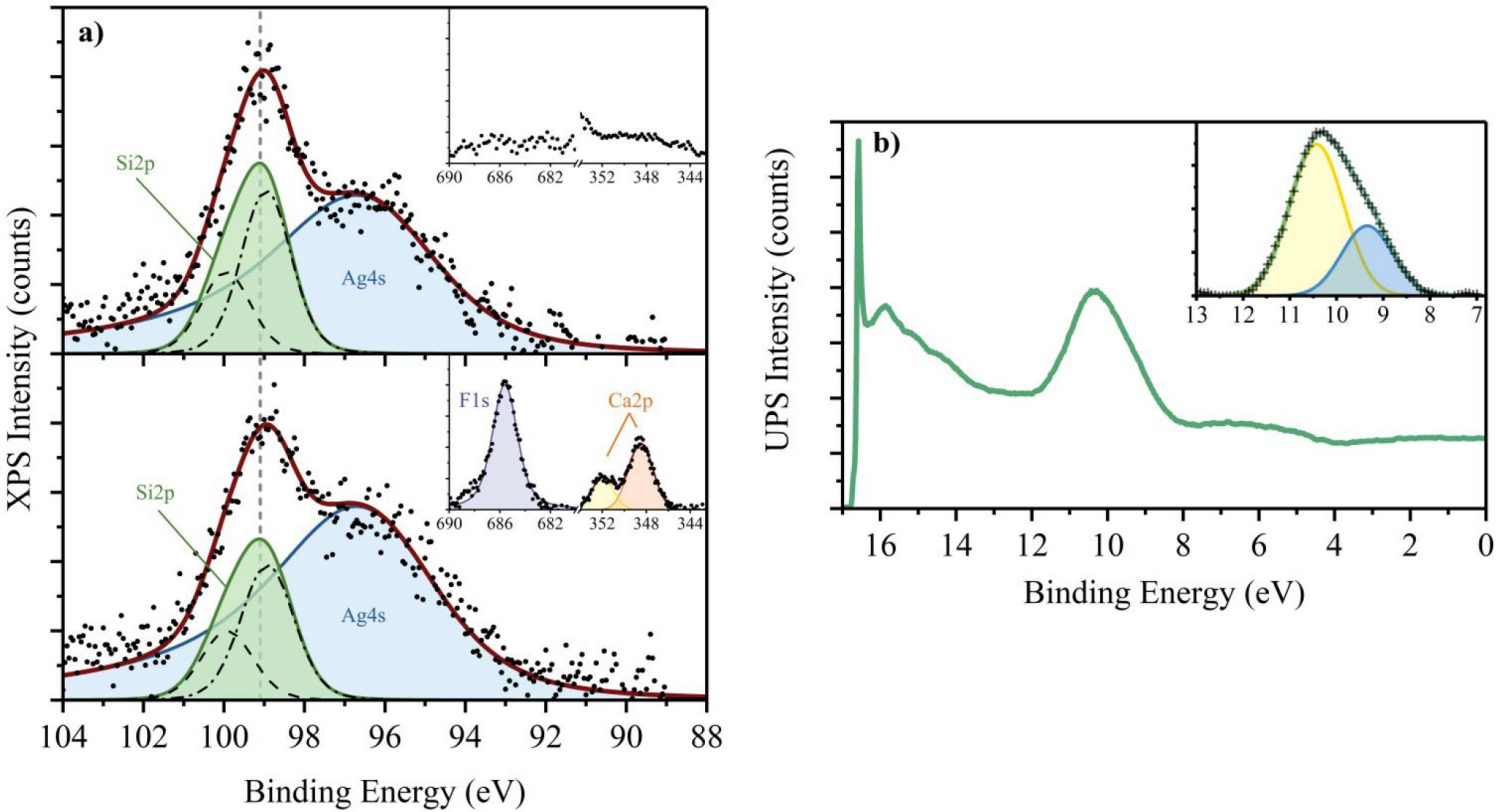
(a) (top) XPS spectrum of the Si 2p peak of 1 ML silicene grown on Ag(111) at 260 °C. The spectrum is composed by the Si 2p doublet (green) and the Ag 4s singlet (blue). (inset) Scan of the F 1s and Ca 2p regions. (bottom) XPS spectrum of the Si 2p peak analyzed after the deposition of the CaF_2_ layer at 260 °C. A scan of the F 1s and Ca 2p regions confirms the presence of CaF_2_, as shown in the inset. The dashed black line indicates the position of Si 2p_1/2_ peak, while the dotted-dashed black line shows the Si 2p_3/2_ one. The dashed gray line corresponds to the position of the combined Si 2p peak. (b) UPS spectrum of 1 nm CaF_2_ deposited on one ML of silicene/Ag(111). The sharp peak at 16.58 eV denotes the onset of secondary electron emission. The broad peak centered at ∼10 eV is related to the valence states of CaF_2_. (inset) Fit shows that the broad peak can be modeled by considering a two-component model.

Figure 2a shows the XPS signal of the Si 2p peak, indicative of the chemical state of the Si atoms in the 2D layer, before (top) and after (bottom) the CaF_2_ deposition onto the silicene/Ag stack. The spectra in the main panels show the Si 2p doublet (green) and the Ag 4s singlet (blue) peaks. Spectra are collected at a takeoff angle of 60°, to improve the sensitivity toward silicene and to reduce the intensity of the Ag 4s related signal, which partially overlaps with the Si 2p peak. The Si 2p peak, accounting for the Si 2p_1/2_ (dashed black line) and the Si 2p_3/2_ (dotted-dashed black line) components, shifts by only 31 meV after the CaF_2_ deposition, likely due to a charge transfer between the CaF_2_ layer and the silicene. It is known that fluorine atoms induce a positive charge on the Si atoms, causing an ∼0.9 eV shift of the Si 2p peak toward higher binding energies upon formation of Si–F bonds,^30,31^ while Ca atoms, being less electronegative than Si, lead to a shift of ∼0.45 eV in the opposite direction.^30,32^ Having observed such a small shift of the Si 2p peak upon CaF_2_ deposition, the formation of covalent bonds between Si and Ca or F can be excluded. In contrast, pronounced shifts of the Si 2p peak were observed in previous experiments where CaF_2_ was grown on regular, sp^3^-hybridized Si and at higher temperatures, strongly suggesting the formation of Ca–Si and F–Si bonds at the interface.^30,33,34^ The insets show the respective XPS spectra at the energy ranges relative to the F 1s and the Ca 2p peaks. After CaF_2_ deposition, the characteristic Ca 2p and F 1s peaks can be observed with a corresponding stoichiometry of 1:2.

To probe the valence band properties of the deposited CaF_2_, UV photoemission spectroscopy is used. The collected spectrum, shown in Figure 2b, is characterized by a narrow peak at 16.58 eV signaling the onset of secondary electrons emission and by a wider peak centered around 10 eV, compatible with the valence states of CaF_2_.^34^ The peak found at lower binding energies can be well-fitted using a two-component model, as shown in the inset. This double-peaked nature of the valence band is typical for bulk alkali halides, and it is predicted by band structure calculations.^35^ The fitted peaks are centered at 9.3 eV and at 10.4 eV, in line with previous results,^34^ showing that the deposited film is in pristine condition.

In contrast to the actual CaF_2_ deposition on silicene at 260 °C, the much higher deposition temperatures (500–750 °C) used for the investigations on Si(111) mentioned above most probably lead to decomposition of CaF_2_ at the interface and the formation of distinct Si–F and Ca–Si bonds.

Furthermore, one should keep in mind that the Si 2p signal in Figure 2a is generated by a bidimensional layer of Si, and therefore it is related exclusively to atoms that are interfacing CaF_2_ without contributions related to bulk material. Since CaF_2_ is completely covering the silicene layer, as deduced from LEED images, all Si atoms are interfacing the CaF_2_ fluoride layer. This means that the formation of a chemical bond, different from the existing Si–Si, would lead to a significant shift of the Si 2p peak and not just a modification of its shape, a condition clearly ruled out by the present data.

The current XPS data thus clearly demonstrate that the chemical state of the Si atoms remains unchanged upon CaF_2_ deposition but do not allow any statements regarding the structure of the silicene layer.

To investigate possible structural modifications of silicene, a thicker CaF_2_ layer (10 nm) was deposited at the same growth temperature of 260 °C, acting as an effective passivation layer, thus enabling ex situ Raman investigations.

**Figure 3:**
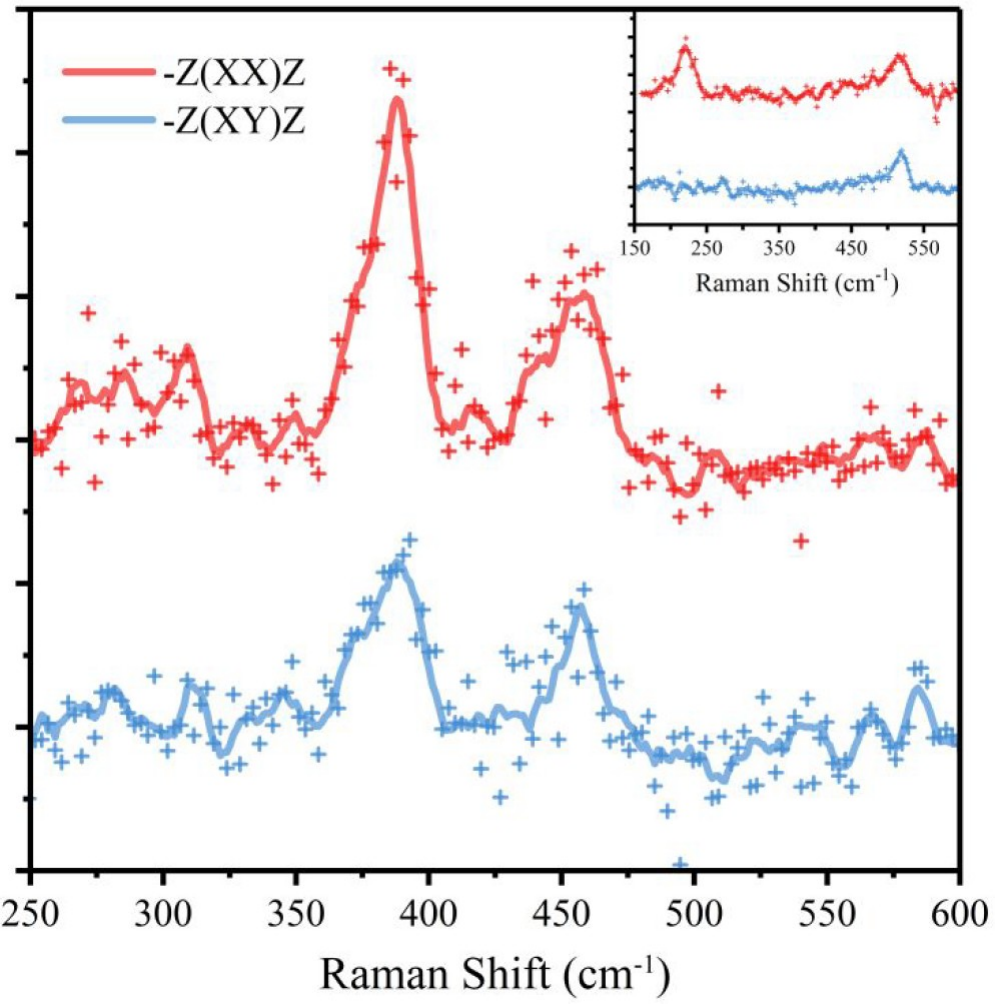
Ex situ Raman spectrum of one ML of silicene grown on Ag(111) at 260 °C and covered by 10 nm CaF_2_. (inset) Raman spectrum of silicene encapsulated under a few-layer graphene flake. In both cases, the spectrum collected in parallel polarization is shown in red, while the one obtained in crossed polarization is in blue.

The polarized Raman spectra for a CaF_2_-covered silicene layer are shown in Figure 3, for parallel (red) and crossed (blue) polarization configurations where the vectors of the incident and scattered light are, respectively, parallel and normal to each other.

Two main Raman peaks are located at 388 and 457 cm^–1^. The observed Raman spectrum deviates strongly from the ones observed for a bare silicene layer on Ag(111), with the characteristic Raman peaks located at 175, 216, and 514 cm^–1^.^17,36^

This drastic change in the Raman spectrum is a clear indication of structural modifications of silicene due to the presence of the CaF_2_ encapsulation. We can correlate the modification in the Raman spectrum to a change in the structure of the 2D layer because the formation of new covalent bonds is excluded by the XPS data. This behavior is completely different to what we have shown when silicene is interfaced with a true van der Waals material, like graphene, where the extremely low out-of-plane forces are not able to change the structure of the 2D material, leaving the Raman signature unaltered.^36^ As a comparison, the spectrum of a graphene-capped silicene is reported in the inset of Figure 3. Note that, in the inset, the peak located at 175 cm^–1^ is not shown due to the limitations of the Raman spectrometer.

A fully van der Waals interaction was also reported in the case of a silicene layer encapsulated between graphene and Ru(0001), but no Raman signature was reported.^37^

Notably, first-principle calculations predict that silicene and CaF_2_ have a pure van der Waals interaction.^38^ However, the vibrational modification presented here is a proof of a stronger interaction between the two materials.

Nevertheless, even small interactions have been reported to be able to change the Raman fingerprint of silicene. Quasi-freestanding silicene obtained through oxygen intercalation shows a modified Raman spectrum even if its Dirac band structure is intact.^39^

When covalent bonds are formed with light hydrogen atoms, the silicene Raman signature is even more heavily perturbed. This happens as a consequence of the increased buckling of the Si atoms, following their rehybridization from a mixed sp^2^-sp^3^ character to a dominant sp^3^ one. The formation of Si–H bonds can be excluded in this work, as hydrogenated silicene is characterized by a totally different Raman spectrum.^40^

When analyzing the structural changes due to the CaF_2_ deposition, it is important to first note that these do not lead to the formation of bulk-like, sp^3^-hybridized Si clusters, as these would generate a sharp and very intense peak located at 520 cm^–1^,^36^ which obviously cannot be observed in the spectra shown in Figure 3. In a crossed polarization configuration (blue spectrum), the intensity of the peak related to the vibrational mode located at 388 cm^–1^ is clearly reduced, while the peak at 457 cm^–1^ is similar in both configurations. This demonstrates that the two observed peaks must be related to phonon modes with different symmetries. Notably, the peak located at 388 cm^–1^ is also observed in CaSi_2_ structures. In these, it is assigned to the out-of-plane vibrations of the Si atoms, which are arranged in 2D, silicene-like planes stacked between Ca planes.^41^ The same peak is also observed in silicene layers obtained by removing Ca from CaSi_2_ through HCl deintercalation, and it is also assigned to the same vibration of the 2D Si planes.^42,43^ The observed polarization dependency and the similarity to the CaSi_2_ spectrum strongly suggest that the 388 cm^–1^ mode is related to the out-of-plane vibration of the Si atoms, with a strong blueshift compared to bare silicene on Ag(111). It is then reasonable to assign the 457 cm^–1^ vibrational mode to the in-plane vibration of Si atoms: this mode is therefore strongly red-shifted, if compared to the one detected in silicene/Ag(111),^17,36^ but blueshifted with respect to the in-plane mode of Si atoms in CaSi_2_ structures.^41^

We note that the out-of-plane (OOP) vibrational mode in silicene structures is activated by the presence of buckling:^17,44^ such a vibrational mode is not visible, for example, in fully planar graphene. It also appears that the frequency of this vibrational mode is correlated with the buckling height of the structure: for freestanding silicene, the predicted buckling is 0.44 Å, and the frequency of the OOP vibrational peak is 175 cm^–1^. For silicene on Au(111), the buckling is strongly reduced (∼0.24 Å), and the OOP vibrational peak is located at 98.6 cm^–1^.^45^ In silicene on Ag(111) the buckling height is expected to be higher (0.8 Å), and the main out-of-plane Raman mode is located at 216 cm^–1^.^17,21^ In CaSi_2_ structures, where the out-of-plane vibrational mode is centered at 388 cm^–1^, the buckling height is even higher (0.92 Å).^46^

The detected changes in the silicene Raman spectrum thus point to an increase of the buckling height of the silicene plane, getting closer to the value observed in CaSi_2_. The increased buckling implies a longer Si–Si bond length, resulting in a red-shifted in-plane (breathing) vibrational mode,^47^ compared to the silicene/Ag(111) case. A schematic representation of the different silicene structures is reported in Figure S2.

It is important to note that, differently from the case of bare silicene on Ag(111), the out-of-plane vibrational mode is not totally suppressed in a cross-polarization configuration. This is a clear indication that, due to structural modifications, the silicene layer is now less symmetric, not belonging anymore to group *C*_6_*_v_*. The local buckling of each Si atom in the unit cell of silicene might have changed, compared to the bare silicene/Ag(111). This deformation, however, does not lead to the formation of bulk-like sp^3^ hybridized Si and comes without the formation of covalent bonds between Si and either F or Ca atoms, as proven by the XPS measurements. CaF_2_ can be therefore employed to trigger a structural modification of silicene, obtaining a layer characterized by a higher buckling. We note that this structural change does not depend on the amount of deposited CaF_2_ but just on the interaction with the capping material. It can be expected that such a structural modification will also have an impact on the electronic properties of the 2D layer.^47,48^ Nevertheless, it has been shown that the Dirac features are preserved even under a high deformation of the silicene layer.^47,49^

As mentioned earlier, the structural modification of silicene could have been triggered by the weak interaction with the fluoride layer. It has been observed that, through the intercalation of F^–^ ions in a CaSi_2_ crystal, silicene bilayers are obtained.^50^ Silicene planes in CaSi_2_ are anionic, due to charge transfer from the Ca atoms. The authors observe that, after F intercalation, every couple of silicene layers assemble to reduce the number of unsaturated bonds. In the present case, however, only one silicene layer is available, impeding the formation of bilayers. More importantly, all the Ca atoms are bonded to the F atoms, as indicated by the XPS analysis of the CaF_2_ layer of Figure 2a, strongly suppressing charge transfer to the Si atoms. Nevertheless, even the small charge transfer detected through the XPS analysis could have triggered the structural reorganization of the silicene monolayer.

We expect the epitaxial growth of CaF_2_ on silicene to be successful independently of the chosen substrate, given that the lattice parameter of silicene is not heavily perturbed by the interaction with the supporting material. Nevertheless, silicene grown on Ag(111) and encapsulated under CaF_2_ can be directly employed in the fabrication of functional devices. This can be achieved by separating silicene from the conductive substrate through O_2_ intercalation^39^ or through a combination of chemical and mechanical steps that enable the transfer of silicene to different substrates or the removal of Ag from the channel area.^51^

## Conclusions

To summarize, we have investigated CaF_2_ on top of a stack composed by one ML of silicene grown on Ag(111) as a promising material for the realization of an effective gate insulation layer in silicene-based electronic devices. LEED patterns prove that the CaF_2_ layer is crystalline and grows epitaxially following the orientation of the silicene phases that form on Ag(111). An XPS analysis shows unambiguously that the Si 2p peak of the silicene layer does not shift upon deposition of CaF_2_, which excludes the formation of covalent bonds between Ca, F, and Si atoms. Polarization-dependent Raman spectroscopy of the buried layer evidences that silicene undergoes structural modifications when interfaced with CaF_2_ but maintains the 2D nature. The shifts of the main Raman components are indicative of an increased Si–Si bond length and of a larger separation between the two buckled planes of Si atoms. Our results demonstrate that silicene can be successfully interfaced with CaF_2_, without losing its bidimensional character. It is important to consider that the structural modification that silicene exhibits may lead to a significant change in the electronic transport properties, which should be carefully assessed in a follow-up study.

## Methods

All the growth experiments were performed in an ultrahigh-vacuum (UHV) system at a base pressure of 5 × 10^–11^ mbar. Single-crystalline layers of Ag(111) on mica (MaTeck GmbH) were used as growth substrates and thoroughly cleaned in situ through cycles of Ar^+^ sputtering and subsequent annealing at 520 °C. Various phases of silicene were grown at substrate temperatures of 260 or 300 °C. The substrate temperature was measured using an infrared pyrometer (DIAS DGE10n) with a precision of ±2 °C.

Silicene growth was achieved by Si evaporation from a rod (Goodfellow GmbH), using an electron beam evaporator (SPECS EBE-1), at a deposition rate of ∼0.02 ML/min. For the actual growth of the insulating layer, CaF_2_ crystals (Sigma-Aldrich, GmbH) were evaporated from a tungsten crucible mounted in another electron beam evaporator (SPECS EBE-4) at a rate of ∼1.2 nm/hour.

An LEED analysis was performed using the SPECS ErLEED 100 optics at an electron energy of 35 eV and at low exposure times to prevent damage to the CaF_2_ layer. XPS and UV photoelectron spectroscopy (UPS) analyses were also performed in situ. Therefore, photons with an energy of 1486.65 eV are generated using an SPECS XR50 source equipped with an Al anode for XPS analysis. For UPS, photons with an energy of 21.2 eV are generated by a He plasma discharge using an SPECS UVS 10 source. The emitted photoelectrons are collected at an angle of 60° in an SPECS 150 hemispherical analyzer and detected by a charge-coupled device (CCD) detector. The acquired spectra are analyzed using the software CasaXPS: first, the background is subtracted recurring to a Tougaard model; peaks are then fitted using modified Lorentzian functions.

Finally, ex situ polarized Raman spectroscopy is performed immediately after removal of the samples from the UHV system, before the silicene layer is destroyed by oxidation. The analysis is performed in a backscattering geometry using a confocal μ-Raman setup (Alpha 300, WITec) equipped with a frequency-doubled (λ = 532 nm) Nd:YAG laser. The intensity of the laser is kept under 0.5 mW, and the exposure time is under 60 s to prevent heating effects. The measured spectrum is smoothed using a Savitzky-Golay filter.

## Supporting Information Available

Schematic representation of the different silicene phases obtainable on Ag(111), Schematic representation of different silicene structures (PDF)

## Abbreviations

LEED: Low Energy Electron Diffraction
MBE: Molecular Beam Epitaxy
XPS: X-ray Photoemission Spectroscopy
UPS: UV Photoemission Spectroscopy
