# Supplementary material for: Epitaxial Growth of Crystalline CaF2 on Silicene
Source: ACS Appl Mater Interfaces. 2022 Jul 6;14(28):32675–82. doi: 10.1021/acsami.2c06293 (PMC9305960; doi:10.1021/acsami.2c06293)
Supplement: Supplementary file 1 — am2c06293_si_001.pdf [file am2c06293_si_001.pdf]

Supplementary Information

Epitaxial growth of crystalline CaF2 on silicene

Daniele Nazzari†,*, Jakob Genser†, Viktoria Ritter†, Ole Bethge§, Emmerich Bertagnolli†, Tibor Grasser#, Walter M. Weber†, and Alois Lugstein†,*

† Institute of Solid State Electronics, Technische Universität Wien, Gußhausstraße 25-25a, 1040 Vienna, Austria

§ Infineon Technologies Austria AG, Siemensstraße 2, 9500 Villach, Austria

Institute for Microelectronics, Technische Universität Wien, Gußhausstraße 27-29, 1040 Vienna, Austria

*Corresponding Authors:

Daniele Nazzari (daniele.nazzari@tuwien.ac.at), Alois Lugstein (alois.lugstein@tuwien.ac.at)

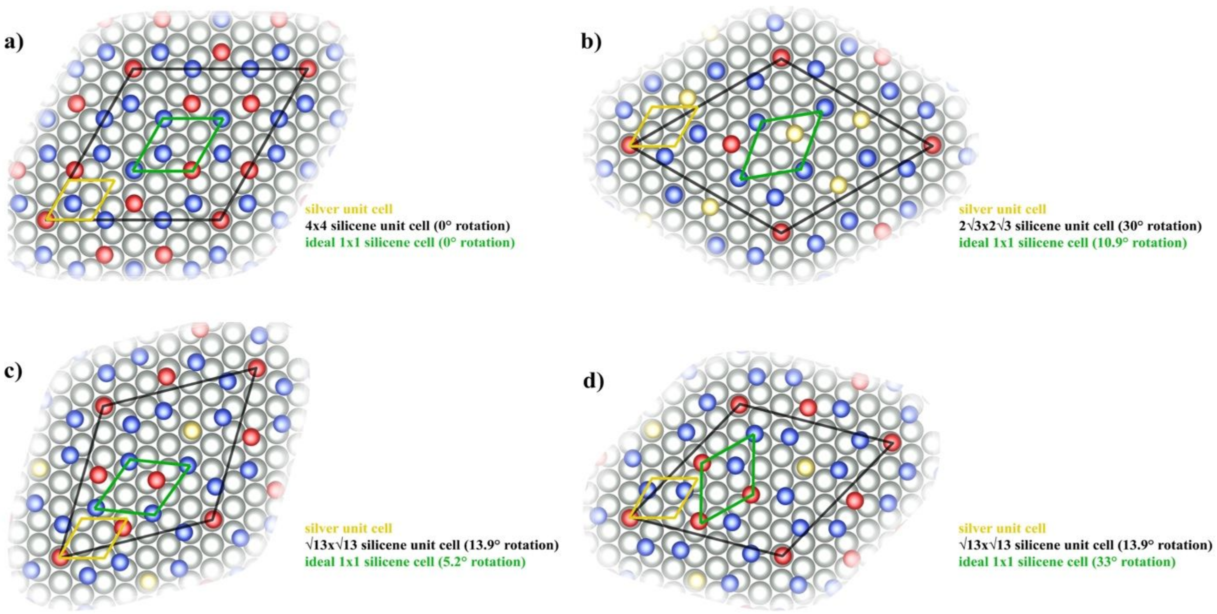

Figure S1. Schematic representation of the different silicene phases obtainable on Ag(111). Grey: silver atoms; blue: silicon atoms with lowest buckling; red: silicon atoms with highest buckling; yellow: silicon atoms with intermediate buckling. a) 4x4 supercell; b) 2√3x2√3R30˚ supercell; c,d) √13x√13R13.9˚ supercell.

Alongside the unit cell of the Ag(111) plane (yellow) and the supercell (black) of the different silicene phases, the unit cell corresponding to the ideal freestanding silicene are also represented (green).

CaF_2 grows following the orientation of the layers represented by the green unit cells. The buckling structure of silicene on Ag(111) has been calculated by Pflugradt et al.[1]

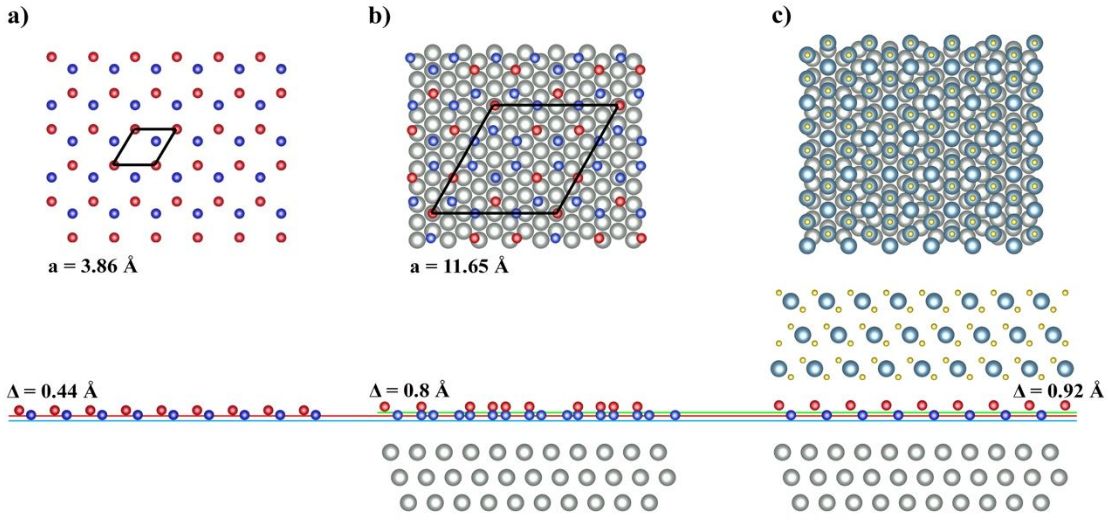

Figure S2. Schematic representation of different silicene structures. a) freestanding silicene, with a buckling of 0.44 Å and a lattice parameter of 3.86 Å. Blue: low buckled Si atoms; red: high buckled Si atoms; b) 4x4 silicene on Ag(111) – the most common phase – with a buckling of 0.8 Å and lattice

parameter of 11.65 Å. This corresponds to the size of the supercell, which is determined by the different buckling of the Si atoms. The actual position of the Si atoms in the plane does not change much from the freestanding case. Grey: Ag atoms; blue: low buckled Si atoms; red: high buckled Si atoms. c) silicene in between Ag(111) and CaF_2, with a buckling value (0.92 Å) matching the one of silicene in $CaSi_2$. The supercell size depends on the precise buckling configuration of each Si atom, an information that cannot be retrieved from the data of the present manuscript. The position of the Si atoms in the plane is very similar to the b) case. The higher buckling determines a longer Si-Si bond. Grey: Ag atoms; blue: low buckled Si atoms; red: high buckled Si atoms, light blue: Ca atoms; yellow: F atoms.

(1) Pflugradt, P.; Matthes, L.; Bechstedt, F. Silicene-Derived Phases on Ag(111) Substrate versus Coverage: Ab Initio Studies. *Phys. Rev. B* 2014, *89* (3), 035403. https://doi.org/10.1103/PhysRevB.89.035403.
